# Morpholino Target Molecular Properties Affect the Swelling Process of Oligomorpholino-Functionalized Responsive Hydrogels

**DOI:** 10.3390/polym12020268

**Published:** 2020-01-26

**Authors:** Eleonóra Parelius Jonášová, Bjørn Torger Stokke

**Affiliations:** Biophysics and Medical Technology, Department of Physics, NTNU—Norwegian University of Science and Technology, NO-7491 Trondheim, Norway; nori.parelius@protonmail.com

**Keywords:** oligo-morpholino-co-Aam hydrogels, responsive hydrogel, interferometric readout

## Abstract

Responsive hydrogels featuring DNA as a functional unit are attracting increasing interest due to combination of versatility and numerous applications. The possibility to use nucleic acid analogues opens for further customization of the hydrogels. In the present work, the commonly employed DNA oligonucleotides in DNA-co-acrylamide responsive hydrogels are replaced by Morpholino oligonucleotides. The uncharged backbone of this nucleic acid analogue makes it less susceptible to possible enzymatic degradation. In this work we address fundamental issues related to key processes in the hydrogel response; such as partitioning of the free oligonucleotides and the strand displacement process. The hydrogels were prepared at the end of optical fibers for interferometric size monitoring and imaged using confocal laser scanning microscopy of the fluorescently labeled free oligonucleotides to observe their apparent diffusion and accumulation within the hydrogels. Morpholino-based hydrogels’ response to Morpholino targets was compared to DNA hydrogels’ response to DNA targets of the same base-pair sequence. Non-binding targets were observed to be less depleted in Morpholino hydrogels than in DNA hydrogels, due to their electroneutrality, resulting in faster kinetics for Morpholinos. The electroneutrality, however, also led to the total swelling response of the Morpholino hydrogels being smaller than that of DNA, since their lack of charges eliminates swelling resulting from the influx of counter-ions upon oligonucleotide binding. We have shown that employing nucleic acid analogues instead of DNA in hydrogels has a profound effect on the hydrogel response.

## 1. Introduction

DNA-based responsive hydrogels are a promising group of materials capable of adapting their properties to the presence of various molecular targets in their environment, ranging from DNA [1,2,3] and other organic molecules [4,5,6], through ions [7,8,9,10] to viruses [11] and cells [12,13]. The mechanism of recognition relies on the ability to synthesize a custom DNA base sequence, which controls the structure of the DNA molecule down to the nanolevel, and on the Watson–Crick complementarity rules that govern the base pairing and interactions with other DNA or non-DNA (via aptamer interactions) molecules. DNA thus offers remarkable sensitivity and specificity in its interactions and the hydrogel provides a means to amplify the molecular signal to a microscopic length scale. The possible applications are in sensing [4,10,14,15], targeted drug delivery [16,17,18], as well as in tissue engineering and as soft devices [1,19,20,21].

**Figure 1:**
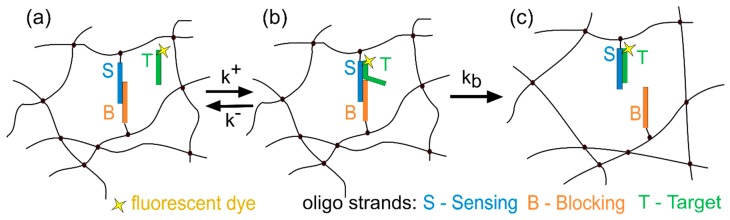
Dual-crosslinked polyacrylamide and nucleic acid (analogue) based hydrogel. In (**a**) the initial state of the crosslink composed of sensing and blocking strand, along with the free fluorescently labelled target. The target can bind (**b**) or dissociate from the crosslink with binding and dissociation rate constants k^+^ and k^−^. Ultimately, (**c**) the blocking strand is entirely displaced after branch migration (k_b_).

One of the DNA hydrogel designs for sensing applications was introduced in 1996 by Nagahara and Matsuda [22] and further investigated in our group [23,24]. The hydrogels in question consist of a dual crosslinked polyacrylamide network (Figure 1), in which the covalently crosslinked polyacrylamide carries oligonucleotide-based physical crosslinks that can be opened in a process of toehold-mediated strand displacement [25]. In short, the oligonucleotide crosslinks are formed by two 5′acrydite-functionalized oligonucleotides that have a complementary region at their 3′ ends. The strands form a partially hybridized duplex and the acrydite allows them to be incorporated through covalent bonds into the hydrogel network as an additional crosslink. This crosslink can be disrupted by the binding of a target oligonucleotide *T*, which is complementary to one of the crosslink strands—sensing strand *S*. Nearly the whole complementarity region between *S* and *T* is blocked by the other crosslink strand—the blocking strand *B*. The bases that are complementary to *T*, but not blocked by *B*, form the toehold—a domain available for the initial binding of the target. The binding is then followed by a migration of the junction point along the length of the *S* strand, until the strand *B* is entirely displaced.

In recent years, synthetic nucleic acids have been explored in preparation of responsive hydrogels [26], in order to overcome some of the drawbacks of native DNA molecules, such as its high charge and subsequent salt dependence or susceptibility to degradation by enzymes. Morpholino oligonucleotides (MOs) are particularly promising due to their uncharged backbone and high solubility. The solubility can be attributed to their well stacked nucleobases. In fact, the stacking is better than that of DNA [27], yielding very good solubility (as an example, 263 mg of a Morpholino 22-mer was dissolved in 1 mL of water without reaching saturation [28]). They are also resistant to nucleases and stiffer than DNA, minimizing self-hybridization. Their melting temperature is slightly higher than that of DNA strands of corresponding sequence. 

Employing MOs instead of DNA in the above described hydrogels provides the advantage of electroneutrality, thus eliminating the electrostatic interactions between the hydrogel and the target. Electrostatic interactions contribute to the partitioning of solutes in gels and thus affect their transport. The partition coefficient is defined as a ratio of the solute’s concentration inside the gel $c_{gel}$ and in the immersing solution $c_{sol}$ at equilibrium:

(1) $$\begin{array}{c} K=\frac{C_{gel}}{C_{sol}}. \end{array}$$

It depends on the size and conformation of the solute and the hydrogel and on their various interactions. If these interactions are considered independent, they can be separated into individual contributions [29]:

(2) $$\begin{array}{c} lnK=lnK_{el}+lnK_{hphob}+lnK_{biosp}+lnK_{size}+lnK_{conf}+lnK_{o}, \end{array}$$

where *el*, *hphob*, *biosp, size*, *conf,* and *o* denote, respectively, interactions of electrostatic, hydrophobic, biospecific affinity, size-related, conformational nature, and other interactions. 

The size and conformation addition to the partition coefficient has been derived by Ogston [30], based purely on hard sphere interactions (i.e., without electrostatic, hydrophobic, and biospecific interactions). The model is derived by placing spheres of radius *a* (solute) in a matrix of long cylindrical fibers of radius $a_{f}$, with a total volume fraction *ϕ*:

(3) $$\begin{array}{c} K_{size,conf}=e^{-\phi {\left(1+\frac{a}{a_{f}}\right)}^{2}} \end{array}$$

In the present study, we extend from the previous investigations employing dsDNA oligonucleotides to MOs as physical crosslinks alongside the covalent ones in the responsive hydrogels. The overall aim is to determine the mutual influence between the various processes, namely target diffusion and binding, physical crosslink disruption, and swelling. A notable difference between the physical crosslinks in MO- and DNA-based hydrogels are the uncharged MOs as compared to the highly charged DNA, implying that one can expect significant differences in the electrostatic contribution to the partition coefficient. The polyanion character of DNA contributes to exclusion of the target from the DNA hydrogel and as a result its slower uptake. Thanks to the electrically neutral backbone of MOs, an increased partitioning is expected, improving the kinetics of the target transport. The MO-polyacrylamide hydrogels are investigated as fabricated on an optical fiber supporting high resolution monitoring of net change in the optical length. This realization also indicates that MO-polyacrylamide hydrogels possess potential as sensing and transducing materials, and although only mRNA sensing proof-of-concept so far has been reported [26], such applications also take advantage of the improved stability of MOs towards enzymatic degradation.

Interferometry and confocal laser scanning microscopy were used to monitor the swelling of the Morpholino hydrogels as well as the uptake of the target within. The swelling was also compared to that of DNA hydrogels of identical nucleotide sequences. 

## 2. Materials and Methods

### 2.1. Materials

Acrylamide ≥ 99% (Aam), *N*,*N*′-methylenebisacrylamide ≥ 99.5% (Bis), squalane oil, dimethyl sulfoxide (DMSO), 3-(trimethoxysilyl) propyl methacrylate 98% (linker), 1-hydroxycyclohexyl phenyl ketone 99% (HCPK) and 2-amino-2-hydroxymethyl-propane-1,3-diol (Tris) were purchased from Sigma-Aldrich; ethylenediaminetetraacetcic acid (EDTA) and sodium chloride (NaCl) were obtained from VWR. Single-stranded Morpholino oligonucleotides and DNA oligonucleotides with custom specified base pair sequence (Table 1) (some functionalized with an acrydite group, some fluorescently labelled at specific base) were obtained from Gene Tools (Philomath, OR, USA) and Integrated DNA Technologies (IDT, Coralville, IA, USA), respectively. All materials were used without further purification. De-ionized water with resistivity 18.2 MΩ cm (Millipore Milli-Q) was used throughout. 

### 2.2. Pregel and Target Solutions

An aqueous buffer prepared from 10 mM Tris, 1 mM EDTA, and 150 mM NaCl, adjusted to pH 7.5 was used for preparation of pregel and solutions containing target Morpholino or DNA oligonucleotides.

Pregel solutions consisted of 10 wt% Aam, 0.6 mol% Bis, 0.13 mol% HCKP, and 0.4 mol% dsDNA or dsMO (duplex SB), dissolved in buffer. HCPK was dissolved in DMSO to the concentration of 0.1 M prior to its addition to the pregel solution. Two different types of hydrogels were prepared—MO hydrogels and DNA hydrogels, differing solely by whether the oligonucleotides incorporated were Morpholinos or DNA. 

Additionally, hydrogels with a different composition were prepared to assess possible radiation damage to DNA in the photoinduced polymerization used. The concentrations of the pregel components were: 6.25 wt% for Aam, 0.2 mol% Bis, 0.6 mol% dsDNA, and 0.2 mol% HCPK, and a labelling strategy using Fluorescein dT on B strand between 12th and 13th base from 5′ end, and dark quencher Iowa Black attached to the 3′ end of strand S was used.

Stock solutions of target ssDNA and ssMO were prepared by dissolving strands *T0*, *T2*, or *T10* in buffer to a concentration of 30 µM. The target stock solutions were prepared from 90% unlabeled and 10% labelled target oligonucleotides (labelled with carboxyfluorescein for MOs, fluorescein for DNA, or for some DNA hydrogels with Alexa Fluor 647, always at the 3′ end). 

### 2.3. Gel Preparation

Quasi-hemispherical hydrogels were prepared at the end face of optical fibers (SMF-28-J9 from ThorLabs, diameter without coating 125 µm) that have been stripped of the coating. The end of the fiber was cut (cutter: Fitel model S323, Furukawa Electric Co. Ltd., Tokyo, Japan), cleaned with ethanol, and functionalized with methacrylate groups by silanization. The silanization procedure consisted of treating the fiber with 0.1 M HCl solution for 20 min and then immersing in a 2 vol% solution of linker in degassed de-ionized water adjusted to pH 3.5 for 15 min. Fibers were again cleaned with ethanol and dust was removed from the end face using duct tape. 

The end of the optical fiber was then immersed in a squalane oil droplet saturated with 2.6 mg/mL of HCPK. A pipette was used to deposit a small amount of the pregel solution (~0.3 nL) at the end face of the fiber. The pregel was polymerized via a free radical polymerization initiated by exposure to UV light for 5 min. The UV source used was a fiber coupled LED UV source M340F3, nominal wavelength 340 nm, ThorLabs, output 813 mW/cm^2^ with an estimated exposure of the pregel of 19 mW/cm^2^ with the employed fiber. Alternatively, for the experiments assessing the radiation damage, a broad spectrum (300–450 nm) UV lamp Dymax Bluewave 50 with an output of 3000 mW/cm^2^ for an equal duration of 5 min was employed. The exposure output is similarly reduced through the fiber as for the LED UV source.

### 2.4. Interferometry

An interferometric readout method described in detail elsewhere [31] was used to monitor the optical length of the hydrogels. In short, a light wave (1530–1560 nm) is sent through the hydrogel which constitutes a Fabry–Perot cavity and the interference of the waves reflected at the fiber-hydrogel and hydrogel-solution interfaces is used to determine the optical length of the hydrogel *L* as well as the change in its optical length Δ*L*. Relative swelling *L%* can be calculated as a change in optical length relative to the initial optical length *L*_0_ (optical length immediately prior to the addition of the target): $L\%\left(t\right)=\Delta L/L_{0}.$ The change in the overall hydrogel swelling volume, *V*/*V*_0_, is related to the change in the optical length by the relation *V*/*V*_0_ ~ [(Δ*L* + *L*_0_)/*L*_0_]^2.6^ where the numerical factor 2.6 differing from three is estimated using finite element analysis of swelling of hydrogels constrained at the optical fiber base [32]. The change in the relative swelling *L%* per unit of time *t:* SR=dL%/dt, was employed as an empirical estimate of the swelling rate. The initial value of this parameter, $SR\left(0\right)={dL\%/dt|}_{t=0}$ was employed as a basis for comparing molecular parameters.

### 2.5. Confocal Laser Scanning Microscopy

**Figure 2:**
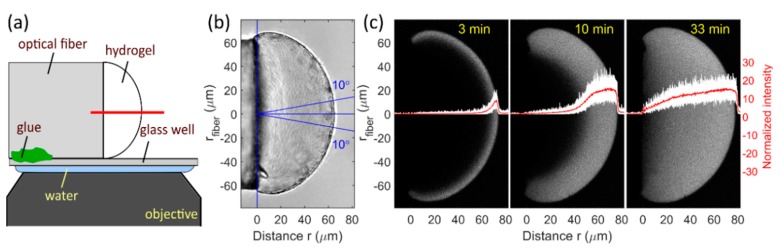
(**a**) schematic illustration of a hydrogel prepared covalently linked to the end face of the optical fiber. The fiber is glued to the bottom of a glass well dish for confocal laser scanning microscopy (CLSM) imaging. The red line indicates the position of the imaging plane (**b**) bright field optical micrograph of a selected MO hydrogel. The lines in blue depict the circular sector from which the fluorescence intensity profiles were extracted and averaged. (**c**) the hydrogel was immersed into a solution of fluorescently labelled target MO-*T10* at time *t* = 0. The CLSM micrographs show the detected fluorescence at times *t* = 3, 10, and 33 min, depicting also in white the individual fluorescence profiles from the circular sector, and in red their average. The same procedure was used for DNA hydrogels.

Confocal laser scanning microscopy (CLSM) was used for monitoring of the spatiotemporal distribution of the target within the hydrogel. For this purpose, the fiber with the hydrogel at its end face was pinched of with tweezers and the fiber was glued to a bottom of a glass bottom microwell dish (P35G-1.5-10-C) from MatTek (Figure 2a). The hydrogel was then left to equilibrate in 100 µL of the buffer solution before 200 μL of 30 μM target stock solution was added immediately before the imaging was started to a final target concentration of 20 μM. 

The imaging was performed using a confocal laser scanning microscope (Zeiss LSM800) with a 40×, NA = 1.2 water immersion objective (optical slice thickness of 0.9 µm) at 22 °C. A micrograph was acquired in a horizontal plane passing through the middle of the hydrogel (depth ~62 µm) every minute, starting 10–20 s after the addition of the target. Excitation wavelength of 480 nm was used for fluorescein with a detection bandpass filter of 500–700 nm. For Alexa Fluor 647, excitation wavelength was 640 nm and the detected fluorescence was filtered by a bandpass filter of 650–700 nm.

### 2.6. Acquiring Relative Concentration Profiles from CLSM Micrographs

Fluorescence intensity profiles were extracted from the CLSM micrographs using custom Matlab R2017a (Mathworks) scripts. Intensity profiles were acquired over several lines from a circular sector spanning 20° around the long axis of the fiber with a 0.5° step. These profiles were then averaged to obtain a smoother fluorescence intensity profile representative of the fluorescence intensity along the axis of the fiber (Figure 2). 

The profile was then smoothed using a Savitzky–Golay filter and normalized so that the intensity of the immersing solution was one (for *T2* and *T10*), or so that the maximum intensity within the hydrogel was one (for *T0*). These fluorescence intensity profiles are referred to as *I_TX_*, where *TX* is one of the targets *T0*, *T2*, or *T10*. 

**Figure 3:**
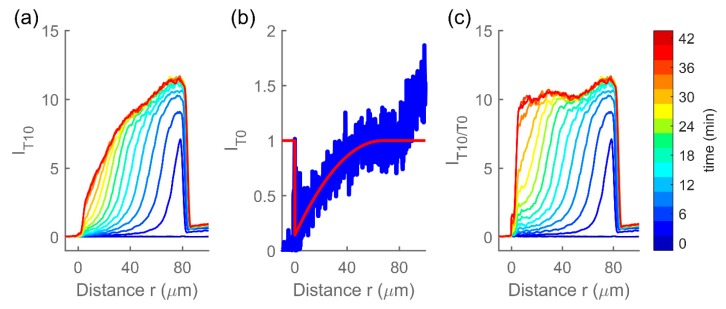
(**a**) an example of fluorescence profiles *I_T10_* recorded by CLSM after addition of target *MO-T10* to a MO hydrogel (angle-averaged, smoothed, and normalized to immersing solution being one). (**b**) the fluorescence intensity *I_T0_* of the same hydrogel immersed in a nonbinding target *MO-T0* in blue and a reference profiled obtained by a second-degree polynomial fit to *I_T0_* shown in red. (**c**) fluorescence intensity profiles *I_T10_* from (**a**) divided by the reference profiles form (**b**), to obtain the relative intensity profiles corrected for the effect of the presence of the fiber, i.e., relative concentration *I_T10/T0_*. Fiber end face is located at *r* = 0.

The close proximity of the glass fiber to the hydrogel affects the detected fluorescence intensity, i.e., the refraction of excitation and emission light through the fiber causes a decrease in the observed fluorescence that is the most pronounced at the fiber end face and reaches as far as 50 µm into the hydrogel/solution [33]. Due to this effect of the fiber, the fluorescence intensity profiles do not reflect the concentration of the target. To correct for this, an intensity profile *I_T0_* in a solution of the non-binding target *T0* was obtained for each individual hydrogel and used as reference for quantifying the effect of the fiber on the fluorescence intensity (Figure 3) [33]. The concentration of the target inside the hydrogel relative to that in the immersing solution can then be obtained by dividing the fluorescence intensity profiles *I_T2_* and *I_T10_* by the reference profile *I_T0_* for each individual hydrogel. The relative concentration profiles are referred to as *I_TX/T0_* where *TX* is one of the targets *T2* or *T10*. 

Relative swelling $R\%=\Delta R/R_{0}$ was also calculated from the obtained relative concentration profiles *I_TX/T0_*. The outer edge of the hydrogel was identified as the position with largest negative slope (obtained by numerical differentiation of the profile), while the inner edge at the fiber end face was identified visually from the micrographs. 

### 2.7. Assessing Possible Radiation Damage

**Figure 4:**
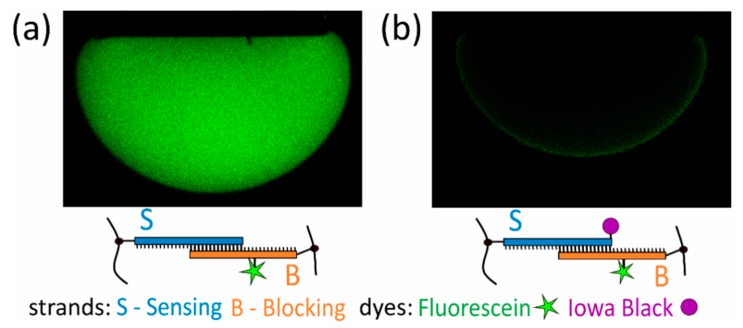
(**a**) A DNA-co-acrylamide (Aam) hydrogel with 10% of the B strands labelled with Fluorescein dT imaged in buffer using CLSM. Fluorescein dT was attached between the 12th and 13th base from 5′ end. Laser power during imaging was 3.5% (**b**) A DNA-co-Aam hydrogel with 10% labelled B strands and 100% S strands carrying a dark quencher Iowa Black at their 3′ ends. Imaged with a laser power of 10%.

Photopolymerization of hydrogels containing DNA has been used previously with various parameters of the UV light exposure, yet possible radiation damage to the incorporated DNA should be considered [2,34,35,36,37]. Possible detrimental effects due to the exposure of the pregel solution containing the SB hybridized dsDNA was assessed using fluorescein dT fluorescent label on 10% of the B strand and a quencher (Iowa Black) on the S strand (Figure 4). Characterization of the hydrogels containing only the fluorescein showed fluorescence intensity throughout the hydrogel (Figure 4a). The finding that practically all fluorescence was quenched in the hydrogel with both the fluorescein and the Iowa Black (Figure 4b) indicates that the proximity between Iowa Black and fluorescein as required for quenching is maintained as designed by the dsDNA. It should be noted that the hydrogels used in these experiments to assess the radiation damage had a different concentration of the pregel constituents than the hydrogels used for the rest of the experiments, namely a lower concentration of both Aam and Bis and higher concentration of dsDNA and the photoinitiator HCPK. As a result, the radiation damage to the DNA in these hydrogels would be expected to be larger than for the hydrogels used in the remainder of the study, due to the larger photoinitiator/Aam and DNA/Aam ratios [38]. Thus, a possible impact of the UVA radiation on the structure of the DNA is not so significant that it disrupted the duplexes. This finding is in line with the data reported by Roh and coworkers [38] and Quick and coworkers [39]. 

### 2.8. Reaction-Diffusion Model

The binding of the target to the hydrogel-bound strands and the subsequent crosslink opening can be modelled as a two-step process characterized by the binding constant *k^+^*, the dissociation constant *k^−^* and the constant of junction point migration *k_b_* (Figure 1). The total molar concentration of available binding sites, i.e., *SB* duplexes available to bind the target is *m_t_*. The concentrations of free binding sites *m_f_*, 3-strand complexes *m_c_*, and open crosslinks *m_o_* add up to the total concentration *m_t_*. Apart from the reaction between the duplex and the target, the target is undergoing diffusion into and through the hydrogel. 

The following partial differential equations for diffusion-reaction in a sphere describe the evolution of the concentration of the free target *c*, as well as the concentrations of binding sites *m_c_* and *m_o_* at a given relative radial position $\hat{r}=r$/R (where *r* is the radial position and R the radius of the sphere) at time *t* [40]. The concentration of target at the boundary is $c_{out}=K\;c_{sol}$ and $\alpha =\frac{D}{R^{2}}>0$.

(4) $$\begin{array}{c} \frac{\partial c}{\partial t}=\frac{\alpha }{{\hat{r}}^{2}}\frac{\partial }{\partial \hat{r}}\left({\hat{r}}^{2}\frac{\partial c}{\partial \hat{r}}\right)-k^{+}cm_{t}+k^{+}cm_{c}+k^{+}cm_{o}+k^{-}m_{c} \end{array}$$

(5) $$\begin{array}{c} B.C.:\frac{\partial c\left(0,t\right)}{\partial \hat{r}}=0,\;t\geq 0 \end{array}$$

(6) $$\begin{array}{c} c\left(1,t\right)=c_{out} \end{array}$$

(7) $$\begin{array}{c} I.C.:c\left(\hat{r},0\right)=\{\begin{array}{c} 0,\;0\leq \hat{r}<1\\ c_{out},\;\hat{r}=1 \end{array} \end{array}$$

(8) $$\begin{array}{c} \frac{\partial m_{c}}{\partial t}=k^{+}cm_{t}-k^{+}cm_{c}-k^{+}cm_{o}-k^{-}m_{c}-k_{b}m_{c} \end{array}$$

(9) $$\begin{array}{c} B.C.:\frac{\partial m_{c}\left(0,t\right)}{\partial t}=0 \end{array}$$

(10) $$\begin{array}{c} m_{c}\left(1,t\right)=0 \end{array}$$

(11) $$\begin{array}{c} I.C.:m_{c}\left(\hat{r},0\right)=0,\;0\leq \hat{r}\leq 1 \end{array}$$

(12) $$\begin{array}{c} \frac{\partial m_{o}}{\partial t}=k_{b}m_{c} \end{array}$$

(13) $$\begin{array}{c} B.C.:\frac{\partial m_{o}\left(0,t\right)}{\partial t}=0 \end{array}$$

(14) $$\begin{array}{c} m_{o}\left(1,t\right)=0 \end{array}$$

(15) $$\begin{array}{c} I.C.:m_{o}\left(\hat{r},0\right)=0,\;0\leq \hat{r}\leq 1 \end{array}$$

This system of partial differential equations can be numerically solved by applying the method of lines and discretizing the spatial dimension to reduce the problem to a system of ordinary differential equations [41,42]. 

The mathematical diffusion-reaction model was then fitted to experimental data, with parameters $\alpha ,\;k^{+},\;k^{-},\;k_{b},\;\mathrm{and}\;m_{t}$ being determined in the fitting. Another parameter, $t_{delay}$, was also determined in the fitting and accounts for the time delay between the addition of the target solution to the hydrogel’s immersing solution and the start of the scanning. The swelling was not considered in the model fitting and the experimental size of the hydrogel has been approximated by its initial radius $R_{o}$. The hydrogels were assumed to be half-spherical in this model. 

## 3. Results and Discussion

### 3.1. Swelling Rate and Equilibrium Depends on the Toehold Length and Differs between DNA and MO

**Figure 5:**
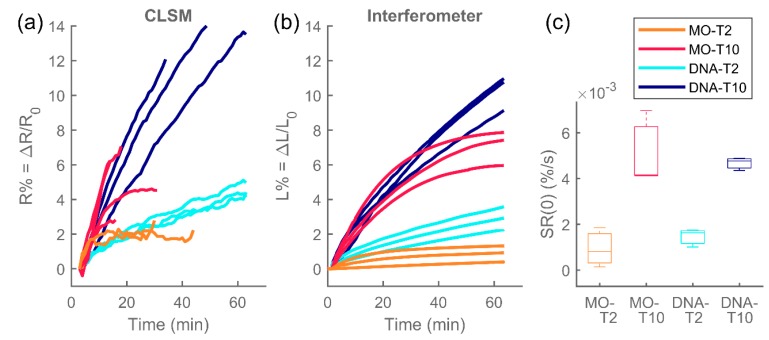
Relative swelling kinetics of Morpholino and DNA hydrogels after exposure to Morpholino and DNA (respectively) targets *T2* or *T10* at *t* = 0. In (**a**), relative swelling as calculated from CLSM micrographs, reflecting changes in the hydrogel’s physical length. In (**b**), relative swelling obtained by interferometry, reflecting the optical length changes. In (**c**), box plots of initial swelling calculated from the interferometer curves. The interferometric and CLSM measurements were conducted on separate hydrogel preparations.

The swelling was monitored both via interferometry and via confocal laser scanning microscopy for separate, parallel hydrogel preparations, i.e., no hydrogels were reused. In Figure 5 relative swelling curves for several parallel hydrogel preparations are shown as measured by the interferometer or the CLSM. The interferometer measures the optical length *L* of the hydrogel with high precision [31], while the physical length R is directly determined from CLSM micrographs with lower precision, (as can also be seen by the CLSM data showing more noise in Figure 5a, compared to smooth interferometer data in Figure 5b). The initial swelling rate at time *t* = 0, SR(0), was calculated from the interferometer data and shown in Figure 5c. 

Similar trends can be observed between interferometric and CLSM data, but the relative optical length change (interferometer) seems to be smaller than the change in the relative physical length (CLSM) which could be attributed to the changes in refractive index, brought on by swelling, but it could also be due to a measurement error, since the edge of the fiber in the micrographs was identified visually and thus introduced a human error and bias. MOs are consistently seen to reach their equilibrium swelling state within or shortly after 60 min, while DNA hydrogels are swelling for several (2–4) h. The equilibrium swelling is also much larger for DNA hydrogels than for the MO hydrogels, while their initial swelling rates differ much less (Figure 5c). While the absolute swelling rates as depicted in Figure 5 are similar for Morpholinos and DNA, the fact that DNA hydrogels reach their equilibrium much later means that their swelling rate as fraction of total swelling change is slower. The limited swelling of MO hydrogels compared to those with DNA can be attributed to the lack of electrostatic interactions, meaning that no influx of counterions (and solvent) accompanies the binding of the MO to the hydrogel. 

There is a significant difference in the swelling rate and the equilibrium swelling depending on the length of the toehold, both in the case of Morpholino oligonucleotides and DNA oligonucleotides. The target *T10* leads to a faster and more pronounced swelling than *T2*.

### 3.2. The Effect of Charges and Fluorescent Dyes on Partitioning

**Figure 6:**
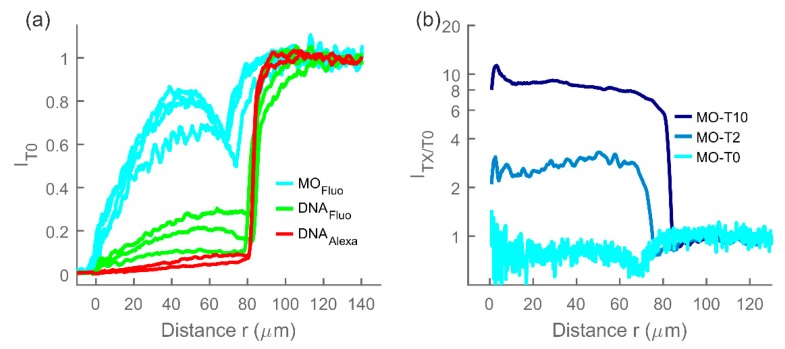
(**a**) normalized fluorescence intensity profiles *I_T0_* of non-binding target *T0* (either MO-*T0* or DNA-*T0*) in MO hydrogels and DNA hydrogels respectively. MOs were functionalized with carboxyfluorescein dye and DNA oligonucleotides were functionalized either with fluorescein or Alexa 647. (**b**) relative concentration profile *I_T0/T0_* of non-binding target *T0*, compared to equilibrium (or near-equilibrium) relative concentration profiles of binding targets *T2* and *T10* (*I_T2/T0_* and *I_T10/T0_*). The individually prepared hydrogels for these data extend up to r = 66 μm, 72 μm, and 82 μm for the nonbinding *T0* and *T2* and *T10* binding targets, respectively.

Fluorescence intensity profiles *I_T0_*, uncorrected for the presence of the fiber, are shown in Figure 6a for non-binding target *T0* (either Morpholino or DNA) in corresponding (Morpholino or DNA) hydrogel. In case of DNA, two different fluorescent dyes, attached at the same location in the nucleotide sequence, were tested: 3′-Alexa Fluor 647 and 3′-fluorescein. Morpholinos were only labelled using 3’-carboxyfluorescein. 

The partition coefficient, calculated for the *T0* targets as the ratio of maximum *T0* fluorescence intensity inside the hydrogel relative to the immersing solution, has been determined for each experiment and averaged for the same targets. The obtained values were K=0.77, 0.22, and 0.06 for carboxyfluorescein labeled MOs, fluorescein-labelled DNA, and Alexa Fluor 647-labelled DNA oligonucleotides, respectively. 

The size contribution to the partition coefficient $K_{size}$ can be approximated by the Ogston formula (Equation (3)). The parameter *a* can be approximated by the radius of gyration of a single stranded DNA of 25 bases to *a* = 3 nm [43] (this value would be somewhat larger for MOs, claimed by others to be stiffer than DNA [26]) and using experimentally determined volume fraction =0.06, and fiber radius $a_{f}=0.8$ nm from Williams at al [44] for polyacrylamide, we would expect the size contribution to the partitioning coefficient to be $K_{size}=0.26$ for DNA and slightly lower for Morpholinos. 

Since Morpholino oligonucleotides have an electrically neutral backbone, the electric potential contribution to the partition coefficient is eliminated as compared to corresponding DNA hydrogels in which the electrostatic repulsion is leading to larger exclusion of the target from the hydrogels. The MOs however appear to have other interactions with the hydrogel, since their partition coefficient is larger than the estimate of the size contribution alone, suggesting less depletion of MOs within the hydrogels. This could be attributed to hydrophobic interactions, as Morpholinos have been reported to exhibit interactions with hydrophobic molecules most likely due to their own hydrophobic nature [45,46].

We can also observe a difference in partitioning for the DNA strands labelled with different fluorescent dyes, namely fluorescein-labeled DNA exhibiting less depletion than Alexa Fluor 647-labeled DNA (Figure 6a). Such an effect on partitioning could be a result of hydrophobic or electrostatic interactions as well as due to the dye’s effect on the size and conformation of the DNA-dye complex. 

### 3.3. Toehold Effect on Target Spatiotemporal Distribution in the MO Hydrogels

In Figure 6b the relative concentration profile of the non-binding MO-*T0* in MO hydrogels is compared to the equilibrium (or near-equilibrium for MO-*T2*) relative concentration profiles of binding targets MO-*T2* and MO-*T10* in MO hydrogels. While the non-binding target’s concentration in the hydrogel is below that of the immersing solution, the binding targets MO-*T2* and MO-*T10* are accumulating to approximately three and nine times, respectively, their concentrations in the immersing solution. This difference between binding and non-binding targets reflects the biospecific interaction between the target and hydrogel-bound oligonucleotides with which they were designed, and which supports their possible applications. The targets with longer toeholds (MO-*T10*) are showing more accumulation at equilibrium than those with shorter toeholds (MO-*T2*), a difference that was also reflected in their equilibrium swelling volumes (Figure 5).

**Figure 7:**
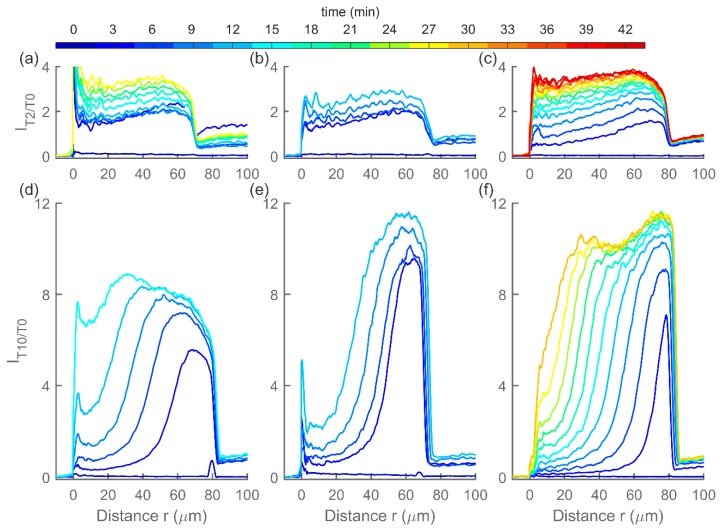
Spatiotemporal evolution of relative target (carboxyfluorescein-labelled MO-*T2* or MO-*T10*) concentrations *I_T2/T0_* and *I_T10/T0_* after exposure of MO hydrogels to the target MO solution at approximately *t* = 0. (**a**–**c**) Spatiotemporal hydrogel response to *T2* for individually prepared hydrogels. (**d**–**f**) Spatiotemporal hydrogel response to *T10* for individually prepared hydrogels. A profile is plotted for every third minute.

Figure 7 shows the spatiotemporal evolution of the MO target in several parallel MO hydrogel preparations as measured by CLSM. Both targets are seen to quickly (within three minutes) accumulate inside the hydrogels to levels higher than those in the immersing solution. MO hydrogels exposed to *T10* have reached saturation at eight–12 times the concentration of the immersing solution, while MO hydrogels immersed in *T2* solution have reached a maximum concentration of three–four times that of the immersing solution. 

A difference in the spatiotemporal distribution pattern can be seen for the two different toehold lengths of the targets. While target *T2* is filling up the hydrogel almost uniformly throughout its volume, target *T10* first saturates the outermost layer of the hydrogel, leading to a sharp concentration change in the hydrogel and this boundary then shifts further towards the center of the hydrogel as each new layer becomes saturated with the *T10* target. This difference suggests that similarly to toehold-mediated strand displacement of DNA [25], the dissociation constant strongly depends on the length of the toehold also for MOs, with the dissociation constant of *T2* being larger than that of *T10*. *T10* binds strongly to the SB duplex and does not readily dissociate, which prevents the targets from penetrating far into the hydrogel before all the available binding sites within the outer layer are occupied. On the other hand, *T2* which dissociates much more easily is quickly released after binding and diffuses further into the hydrogel before another binding occurs. 

**Figure 8:**
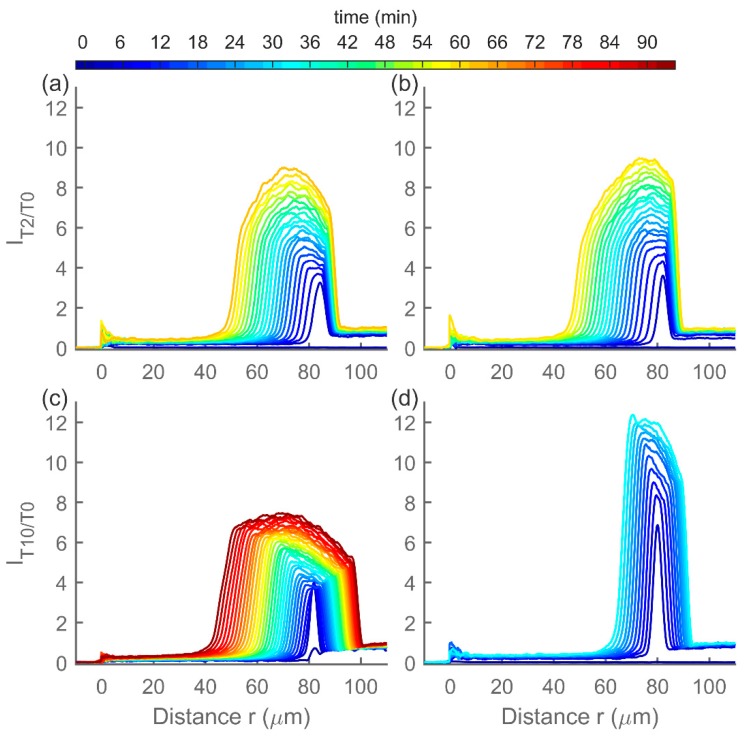
Spatiotemporal evolution of relative target (fluorescein labeled DNA-*T2* or DNA-*T10*) concentrations *I_T2/T0_* and *I_T10/T0_* after exposure of DNA hydrogels to the DNA target solution at approximately *t* = 0. (**a**,**b**) Spatiotemporal hydrogel response to *T2* for individually prepared hydrogels. (**c**,**d**) Spatiotemporal hydrogel response to *T10* for individually prepared hydrogels. A profile is plotted for every third minute. Note that the time scale is different than that in Figure 7.

DNA hydrogels were also exposed to DNA targets (DNA-*T2* and DNA-*T10*) and imaged using CLSM (Figure 8). Compared to MOs, DNA hydrogels are swelling slower and exhibit a steeper concentration gradient moving inside the hydrogels, both in case of *T2* and *T10* targets. Unexpectedly, the concentration is seen to be increasing in the part right of the moving wavefront also for DNA-*T10*, instead of quickly reaching a plateau, as was observed in the case of MOs. This behavior cannot be explained by the association, dissociation, and crosslink opening processes as described in the reaction–diffusion model and suggests another phenomenon taking place in these hydrogels. Additional experiments mapping effects of toehold molecular parameters are also reported elsewhere [24,42]. However, an explicit comparison between the various sets of data is challenging due to differences in concentrations of immobilized dsDNA crosslinks and possible effects of the various fluoroprobe labels.

### 3.4. Estimating Target MO-T10 and MO Hydrogel Properties from Diffusion-Reaction Modelling 

We have fitted the obtained relative concentration profiles for MO-*T10* targets in MO hydrogels (*I_T10/T0_*) to the reaction-diffusion model described in Methods. The fitting was less reliable for MO-*T2* hydrogels and the optimization yielded local minima depending on the starting conditions. These results are therefore not included. 

**Figure 9:**
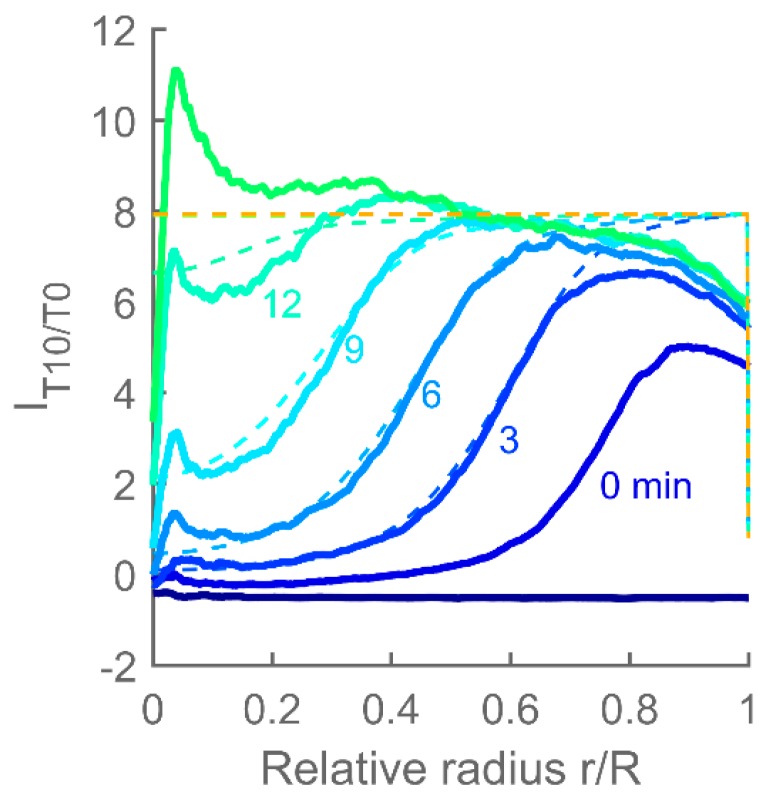
Spatiotemporal evolution of relative target (carboxyfluorescein-labelled MO-*T10*) concentration *I_T10/T0_* after exposure of an MO hydrogel to the target solution at approximately *t* = 0. The depicted hydrogel is the one in Figure 7d. A profile is plotted for every third minute. In dotted lines, the profiles obtained by fitting the numerical reaction-diffusion model to the experimental data.

An example of the model fitted to the experimental data is shown in Figure 9. Fitting was repeated for all the MO hydrogels in MO-*T10* target shown in Figure 7b and the obtained fitting parameters were averaged to estimate the characteristic properties of the MO-*T10* target and MO hydrogels (Table 2). The table also contains the theoretical values of association and dissociation rate constants as modelled by Zhang and Winfree for DNA in solution [25]. The theoretical value for diffusion coefficient is also estimated for a DNA oligonucleotide: DNA of 25 bases has a diffusion coefficient in solution of approximately 130 µm^2^/s [47], which is expected to be reduced to approximately to 16% of its value when in a hydrogel [48], giving value of approximately 21 µm^2^/s. The theoretical concentration of the binding sites is estimated from the concentration of SB duplexes in the pregel solution, which is 5.64 mM, but due to the initial swelling of the polymerized hydrogels, this is reduced to approximately 4 mM at the beginning of the experiment. 

Morpholino hydrogels are showing a smaller diffusion coefficient than estimated for DNA of the same length in a hydrogel of this composition. This could be accounted for by the reported higher stiffness of Morpholinos, but it could also suggest that the attached fluorescent dye affects the diffusion [49]. Concerning the apparent concentration of the binding sites, the hydrogels showing saturation at 0.18 mM suggests that only a small fraction (≈ 5%) of the duplexes are available for binding the target. 

As seen in Table 2, the rate constants also differ significantly from those for DNA in solution, suggesting that the kinetics of toehold exchange is affected by the changes to the backbone and that data estimated for DNA cannot be directly applied to Morpholinos, despite having the same base sequence.

### 3.5. Implications of Differences between MO and DNA Hydrogels

Overall, the data indicate a consistent correspondence between the target MOs being less strongly bound to the embedded MOs and thereby less retarded in their diffusion into the hydrogels as compared to ssDNA targets invading dsDNA-co-Aam gels with the same toehold and blocking length parameters. Correlating this with differences in MO and DNA structures (i.e., identical base sequences with differing backbones) indicates that particularities of e.g., charge nature, hydrophobic/hydrophilic balance play a role when embedded in hydrogel structures as used here. Determination of the specific mechanisms through which these oligonucleotide properties lead to the observed MO–DNA differences would require further investigation.

Based on the current findings and integrated with appropriate readout platforms, molecular design principles exploiting Morpholinos embedded in hydrogels represent a possible innovation route within biosensing. This will additionally require selection of bp sequences of the Morpholinos tailormade for the biomarker, of which microRNA are the most obvious candidates, e.g., as indicated in the recent work in the group of Shaver [26]. A sensitivity of the interferometric readout platform as employed in the present study, showing a 1% change for the MO-*T2* design of the target at 20 μM, can be expected to provide readable signals down to about 200 nM for a biomarker with similar association constant to the embedded dsMO. Biomarkers with larger association constant can be determined at lower concentrations, and we have previously reported a limit less than 10 nM for DNA oligomers [23]. 

Morpholinos are exhibiting a lack of interactions with protein [50], suggesting they are not suitable for development of protein-binding aptamers and as of today, no Morpholino aptamers have been reported (whether targeting protein or other molecules). However, nucleic acid-based hydrogels can be prepared with a multitude of designs, featuring interactions of several oligonucleotides and other molecules [51]. MOs could be employed in such schemes in hybrid configurations together with DNA strands, where DNA strands provide aptamer functionality and MOs can serve e.g., as blocking strands which are displaced by a biomarker specific for the aptamer. Such hybrid systems could be of interest in order to better tune the hydrogel properties but require a thorough investigation first. As was reported in this paper, MOs display significant differences from DNA in their properties and interactions when integrated into a hydrogel.

## 4. Conclusions

Nucleic acid analogues, such as Morpholino oligomers, provide opportunities to exploit the specificity and sensitivity of DNA interactions within hydrogels, while altering other properties of the molecule. Here we have employed Morpholino oligonucleotides as physical crosslinks within polyacrylamide hydrogels and compared their response to target MOs with the response of DNA hydrogels to corresponding DNA targets. Due to the uncharged backbone of MOs, we observed less depletion of non-binding targets compared to DNA, and improved kinetics for binding targets, as well as a less pronounced total swelling response. The dependence of the kinetics on the length of the toehold, known from DNA, was also observed for MOs. Lastly, by fitting the data to a reaction–diffusion model, we estimated the diffusion coefficient and the association, dissociation, and branch migration rate constant for MO-*T10* and found that they differ significantly from those predicted for DNA. 

## Figures and Tables

**Table 1:** Oligonucleotide sequences of the sensing, blocking, and target strands, with the position of acrydite groups (Acr) shown and the nucleobases complementary with sensing strand highlighted. Each sequence was realized both as Morpholino oligonucleotide (MO) and DNA oligonucleotides.

Name	Sequence	#Bases
*S*	3′ C GTA AGT AAC TAT CGA CTT CAG TCG TCA-Acr 5′	28
*B*	5′ Acr-TTC AGT CGT CAG CAT TCA TTG ATA GGA C 3′	28
*T2*	5′ G CAT TCA TTG ATA GCT AAT GAC ATA 3′	25
*T10*	5′ G CAT TCA TTG ATA GCT GAA GTC AGA 3′	25
*T0*	5′ TAT CGT AGC AGG CTA CAG GAC TCA A 3′	25

**Table 2:** Parameters obtained by fitting the reaction–diffusion model to the CLSM data for MO-*T10* spatiotemporal distribution in MO hydrogels.

Parameter	Average Value (of 3) ± Standard Deviation	Theoretical Values
Diffusion coefficient *D* (µm^2^/s)	10 ± 2	21 ^(a)^
Association rate constant *k*^+^ (M ^−1^s^−1^)	10^3.2 ± 0.2^	3 × 10^6 (b)^
Dissociation rate constant *k*^−^ (s^−1^)	0.947 ± 0.06	6 × 10^−6 (b)^
Branch migration constant *k*_b_ (s^−1^)	3.1 ± 0.4	2 ^(b)^
Available binding site concentration *m*_t_ (mM)	0.18 ± 0.03	4 ^(c)^

^(a)^ Estimate for DNA of same length in polyacrylamide hydrogels [47,48]; ^(b)^ Zhang and Winfree for DNA in solution [25]; ^(c)^ Concentration of SB duplexes estimated from initial concentration in the pregel.
